# Real-World Effectiveness Following Benralizumab Use in Patients with Severe Eosinophilic Asthma in Romania: A Retrospective Cohort Study (BREEZE)

**DOI:** 10.3390/jcm15020425

**Published:** 2026-01-06

**Authors:** Claudia Lucia Toma, Gabriela Teodorescu, Florin-Dumitru Mihălţan, Stefan Frent, Selda Ali, Mihaela Trenchea, Ancuța-Alina Constantin

**Affiliations:** 1 Department of Pulmonology, Carol Davila University of Medicine and Pharmacy, 020021 Bucharest, Romania; claudia.toma@umfcd.ro (C.L.T.); florin.mihaltan@umfcd.ro (F.-D.M.); ancuta-alina.constantin@umfcd.ro (A.-A.C.); 2 AstraZeneca Pharma, 013714 Bucharest, Romania; gabriela.teodorescu@astrazeneca.com; 3 Centre for Research and Innovation in Precision Medicine of Respiratory Diseases, Department of Pulmonology, University of Medicine and Pharmacy Timisoara, 300041 Timisoara, Romania; 4 Department of Allergology, Carol Davila University of Medicine and Pharmacy, 020021 Bucharest, Romania; 5 TB Dispensary, Ambulatory of the Medgidia Municipal Hospital, 905600 Medgidia, Romania; mtrenchea79@gmail.com

**Keywords:** severe eosinophilic asthma, benralizumab, real-world data, asthma control, Romania

## Abstract

**Background/Objectives**: The outcomes of biologics in severe eosinophilic asthma (SEA) in real-world settings are less known. We describe the SEA population, treatment patterns, and outcomes following benralizumab authorization in Romania. **Methods**: BREEZE was a retrospective chart review study with a pre–post design conducted in five Central Eastern European and Baltic countries, including Romania (July 2022–January 2023). Adult SEA patients receiving ≥1 benralizumab dose in routine care were enrolled with up to 56 weeks (W) follow-up after benralizumab initiation. Using a funnel approach, the number of patients decreased throughout the follow-up; changes from baseline were tested in patients with available data. **Results**: The Romanian cohort included 131 patients (mean age: 54.4 years at benralizumab initiation; 66% females). Half of patients (53%) received 8 benralizumab doses; only 3 discontinued treatment. At benralizumab initiation, 15% were on maintenance oral corticosteroids (mOCS, median dose: 12.5 mg/day prednisone-equivalent; 17/20 patients > 5 mg/day). At W48, 11.4% of 70 patients with available data continued using mOCS (median dose: 5 mg/day; 3/8 > 5 mg/day). The annualized exacerbation rate was 2.61 (95%CI: 2.28–2.98) at baseline, reducing by 89% at W16 and 90% at W48. Blood eosinophils decreased early from a median of 620 cells/μL (94/120 > 400 cells/μL) at baseline to 1 cell/μL at W16 (*n* = 36; *p* < 0.001). FEV_1_ increased from 1.8 L at baseline to 2.06 L at W16 (*n* = 59; *p* < 0.001), 2.15 L at W24 (*n* = 51; *p* < 0.001), and 1.96 L at W48 (*n* = 31; *p* = 0.002). Most patients had poorly controlled asthma (103 with ACT < 16) at baseline; score increased >9 points at W16 (*n* = 81; *p* < 0.001), W24 (*n* = 80; *p* < 0.001), and W48 (*n* = 55; *p* = 0.002). **Conclusions**: Our national cohort contributes to the increasing evidence on the meaningful results of benralizumab in SEA patients treated in routine practice.

## 1. Introduction

Recent estimates regarding asthma prevalence in Eastern Europe report a total of 4.9 million cases (2622 per 100,000) and 1558 (0.46 per 100,000) deaths in 2021. In Romania, the number of prevalent cases accounted for 791,569 (4527 per 100,000), and the number of deaths reached 229 (0.59 per 100,000) [1]. Eosinophilic asthma, one of the severe asthma phenotypes, represents approximately one-quarter (26%) of all asthma cases across Europe, and 4.9% in Romania. This notably lower prevalence in Romania may be due to differences in diagnostic practices or a lack of centralized, nationwide reporting [2].

Severe eosinophilic asthma (SEA) is a clinically challenging subtype due to the symptom burden, being frequently associated with asthma exacerbations requiring emergency room visits and hospital admissions, high-dose chronic corticosteroid use, and suboptimal treatment response [3]. Despite receiving adequate high-intensity treatments with inhaled corticosteroids (ICSs), and add-ons such as long-acting β-agonists (LABAs), leukotriene receptor antagonists (LTRAs), and long-acting muscarinic antagonists (LAMAs), many SEA patients experience persistent symptoms or recurrent exacerbations [3,4]. Up to 10% of patients are refractory to high-dose inhaled therapy, requiring recurrent use or maintenance treatment with oral corticosteroids (mOCS) with potential subsequent dependency, reduced efficacy, and significant side effects [5,6,7].

Advancements in understanding the biology of eosinophilic disease have led to the development of targeted biologic treatments, changing the course of the disease for treatment-resistant SEA patients [8]. The current eosinophil-targeted therapies specifically designed for SEA could be classified by their mechanisms of eosinophil reduction through binding to specific targets with downstream effects: IL-5, and its further neutralization (mepolizumab and reslizumab); IL-5 receptor alpha, with induction of eosinophil apoptosis through antibody-dependent cytotoxicity (benralizumab); IL-4 receptor alpha, with IL-4 and IL-13 signal blocking (dupilumab); and the cytokine thymic stromal lymphopoietin, disrupting its activation (tezepelumab). In addition, through blockade of the immunoglobulin E (IgE) pathway, omalizumab lowers free IgE levels and the expression of the high-affinity receptor for IgE (FcεRI) on basophils and mast cells, decreasing peripheral blood and sputum eosinophil counts [3,8]. The efficacy of these biologic agents relies on data from registration clinical trials, whereas their effectiveness within real-world settings remains an important area of clinical research.

In this paper, we will focus on the real-world use of benralizumab for SEA. This anti-IL-5 receptor biologic is indicated as an add-on maintenance treatment in adult patients with SEA inadequately controlled despite high-dose ICSs and LABAs therapies [9]. Following its authorization, a global real-world study program named XALOC-1 was implemented to assess its effectiveness following administration to SEA patients in routine clinical practice. Across the XALOC-1 program [10], effectiveness data across various geographies have been reported, including Italy (ANANKE study) [11], Portugal (BETREAT study) [12], the United Kingdom (UK, BPAP study) [13], Spain (ORBE-II study) [14], and the Central Eastern Europe and Baltic Area (CEE-BA) region (BREEZE study) [15,16]. While the BREEZE study enrolled 381 patients across five CEE-BA countries, this publication focuses exclusively on the subgroup of patients from Romania. We aim to provide a detailed overview of local clinical practice patterns, disease characteristics, and treatment outcomes within the Romanian population. This national-level perspective may facilitate better alignment with local healthcare policies and may support more targeted decision-making in clinical and public health settings.

## 2. Materials and Methods

### 2.1. Study Design and Patients

BREEZE was a multi-center, observational, retrospective cohort study with a pre-post design conducted in 5 CEE-BA countries (Bulgaria, the Czech Republic, Hungary, Lithuania, and Romania) between July 2022 and January 2023. Following the authorization of benralizumab in January 2018 in Europe [9], reimbursement decisions became effective between July 2019 and June 2020 in the CEE-BA countries. Additionally, a Named Patient-Based Access Program (NBP) was implemented in Romania in November 2019, enabling the real-world use of benralizumab prior to its reimbursement, effective on June 2020. Overall study design, details on the recommended dose of benralizumab, and results from CEE-BA countries have been previously published [16].

In Romania, SEA patients were enrolled consecutively during their regular visits at 23 hospitals and outpatient public and private practice sites distributed across the country. The start date of benralizumab treatment was referred to as the “index date.” The pre-index period covered the 12 months prior to the first benralizumab injection, whereas the post-index period (follow-up) spanned 56 weeks after, with a maximum of nine doses administered during this timeframe. All other standard treatments for SEA followed routine clinical practices.

SEA patients were eligible for inclusion in the study if they were at least 18 years old and had received at least one injection of benralizumab after local treatment authorization, as add-on therapy, as per approved indication in Europe. The list of reimbursement criteria for benralizumab use across Romania is included in the Supplementary Materials. The only exclusion criterion was prior administration of benralizumab or other targeted biologic agents during a clinical trial. Eligible patients were enrolled in the study, regardless of whether benralizumab therapy was ongoing or discontinued at the time of the study initiation, provided they expressed written informed consent to participate in the study. In case of eligible patients no longer under the investigator’s care at the time of study start, a waiver of informed consent was requested for data collection in an anonymized manner, and the Ethics Committee approved this waiver. This approach aimed to enhance generalizability and reduce the bias that would have been generated by including only patients with longer follow-up periods and more favorable outcomes.

The study was conducted according to the principles of the Declaration of Helsinki, the Good Pharmacoepidemiology Practices guidelines of the International Society for Pharmacoepidemiology, and the regulations and guidelines governing medical practice and ethics in Romania. Ethical approval was granted by the National BioEthics Committee for Medicines and Medical Devices in Romania (Comisia Națională de Bioetică pentru Medicamente și Dispozitive Medicale; approval number: 16SNI/30 June 2022).

### 2.2. Study Objectives

The primary objectives were to describe the baseline demographic and clinical characteristics of SEA patients who initiated benralizumab therapy in day-to-day practice, as well as to describe real-world treatment patterns of SEA at baseline and after benralizumab treatment initiation.

Secondary objectives were to describe clinical outcomes at week 16 (W16), week 24 (W24), week 48 (W48), and week 56 (W56) after the initiation of benralizumab in SEA patients.

Exploratory objectives were to describe changes in biomarkers and lung function at W16, W24, W48, and W56 after benralizumab treatment initiation.

### 2.3. Data Collection

Data were collected from paper and/or electronic medical records of each patient by study physicians, then entered into a password-protected, web-based electronic data capture system. The following information was recorded at baseline (during the pre-index period and/or at index date):

- Demographic characteristics: age, sex, smoking history, medical history including key asthma- and OCS-related comorbidities; coronavirus disease 2019 (COVID-19) information.
- Disease and clinical characteristics: frequency and rate of exacerbations (overall and those leading to emergency department [ED] visits, hospitalizations, and systemic corticosteroid use for at least 3 days or temporary increase in stable mOCS use); laboratory parameters (if available): blood eosinophils (bEOS), fractional exhaled nitric oxide (FeNO) level, and total immunoglobulin E (IgE); lung function measurements, if available (forced expiratory volume in 1 s [FEV_1_]), and asthma control test (ACT) scores; healthcare resource utilization in the last 12 months prior to benralizumab start.
- Healthcare resource use: unscheduled visits to general practitioners and specialists, ED visits and hospitalizations, including intensive care unit (ICU) admissions.
- Prior SEA treatments, including biologics: type and duration.

During the post-index period, the following data were recorded at each benralizumab injection visit (if available), corresponding to the schedule used in real-life settings: benralizumab use (duration, discontinuation, reason for discontinuation), mOCS use, concomitant respiratory treatments, frequency and rate of asthma exacerbations, COVID-19 information, healthcare resource utilization, laboratory and spirometry parameters, and ACT scores. A window of ±4 weeks was allowed for the assessment of clinical outcomes, biomarkers, and lung function at each timepoint. Injection visits at W16/W24/W48/W56 corresponded to the doses 4/5/8/9, respectively.

Data were collected until the administration of the last routine benralizumab dose within the maximum 56 weeks of the pre-defined follow-up period, with a maximum 1-week window allowed, or until treatment discontinuation, if discontinuation occurred earlier during this timeframe.

### 2.4. Statistical Methods

All study-eligible patients were included in the full analysis set (FAS). Data were descriptively analyzed and reported based on their distribution. Normally distributed variables were tabulated using the mean with standard deviation (SD), whereas non-normally distributed variables were tabulated using the median with interquartile range (IQR). Categorical variables were tabulated using frequency and proportions. Data were analyzed based solely on observed cases. Missing data were not replaced.

Exacerbation rates and 95% confidence intervals (CI) were calculated using generalized linear regression with a negative binomial distribution. The annual exacerbation rate (AER) was calculated as the total number of exacerbations multiplied by 365.25 and divided by the total duration of follow-up within the sample (days). The duration of baseline mOCS and biologic treatments was calculated as the time (days) between the initiation and discontinuation of treatment; the median duration of treatment (95% CI) was estimated using the Kaplan–Meier method.

The statistical significance of changes from the baseline and/or index date was evaluated using either the paired *t*-test or the Wilcoxon signed-rank test. For multiple comparisons, adjustments were made by applying the Bonferroni correction when appropriate, particularly to address changes from the index.

R language (https://www-r-project.org/; version RStudio 2023.03.1 + 446 “Cherry Blossom”) was used to perform statistical analyses.

## 3. Results

### 3.1. Baseline Patient and Disease Characteristics

One hundred thirty-one patients were included in FAS. At the index date, the mean age (SD) was 54.4 (12.4) years, with 75.6% of patients under 65 years old and 65.7% being female. More than two-thirds (67.9%) were never smokers. The mean (SD) duration of asthma since diagnosis was 11.6 (11) years, with a median of 8 years. The majority of patients (90.8%) experienced at least one asthma-related comorbidity, most frequently chronic rhinitis and allergies. Nearly half (43.5%) had at least one OCS-related comorbidity, with systemic hypertension and cardiovascular disease being the most common (Table 1).

Before benralizumab treatment initiation, the median (IQR) bEOS count was 620 (370–1031) cells/mm^3^, with 72.3% of 130 patients having ≥400 cells/mm^3^. The median IgE level was 143 IU/mL (68–445) among 61 patients with available results. Only 2 patients had FeNO measured at baseline. The median (IQR) FEV_1_ was 1.8 (1.3–2.4) L, based on data available for 108 patients. Uncontrolled asthma was described by 80.5% of 128 patients with available ACTs and scores < 16. Most patients (88.6% of all 131 patients) reported at least one exacerbation in the previous 12 months before benralizumab initiation, with an overall AER (95% CI) of 2.61 (2.28–2.98) (Table 2).

A total of 27 (20.6%) patients reported chronic OCS use in the 12 months prior to initiating benralizumab, with a median (IQR) dose of 15 (10–20) mg/day prednisone-equivalent, and 23 patients (85.2%) receiving high-doses (>5 mg daily). At baseline, before starting benralizumab, all patients used ICS/LABA, 65.7% LTRA, and 34.4% LAMA. Biologics were used by only 9 (6.9%) patients, all of whom had received omalizumab. More details on the treatments used before benralizumab initiation are included in Table 2.

### 3.2. Treatment Patterns During Benralizumab Therapy

At the time of study enrollment, 124 patients (94.7%) were still receiving benralizumab treatment. The median (IQR) number of administered injections was 8 (5–9). Throughout the 56-week follow-up period, half of patients received 8 doses (*n* = 70 [53.4%]) of benralizumab. Three patients discontinued the treatment within the pre-defined follow-up period, of whom one case was due to patient preference/convenience, whereas two other patients changed domicile and moved abroad.

The rate of patients receiving mOCS and other respiratory treatments during benralizumab therapy is shown in Table 3.

At index date, mOCS was ongoing for 20 of 131 (15.3%) patients with a median (IQR) dose of 12.5 (9.4–16.3) mg/day prednisone-equivalent. Of these, 17 (85.0%) were receiving high doses (>5 mg daily). At W16, of the 116 patients with available results, 16 (13.8%) were still using mOCS at a median (IQR) dose of 5 (5.0–10.0) mg/day prednisone-equivalent, with 6 of them (37.5%) receiving >5 mg daily. At W24, of the 106 patients with available results, 13 (12.3%) were still using mOCS at a median (IQR) dose of 5 (5.0–10.0) mg/day prednisone-equivalent, with 6 of them (46.2%) receiving >5 mg daily. At W48, of the 70 patients with available results, 8 (11.4%) were still using mOCS at a median (IQR) dose of 5 (5.0–6.5) mg/day prednisone-equivalent, with only 3 (37.5%) receiving >5 mg daily.

**Figure 1:**
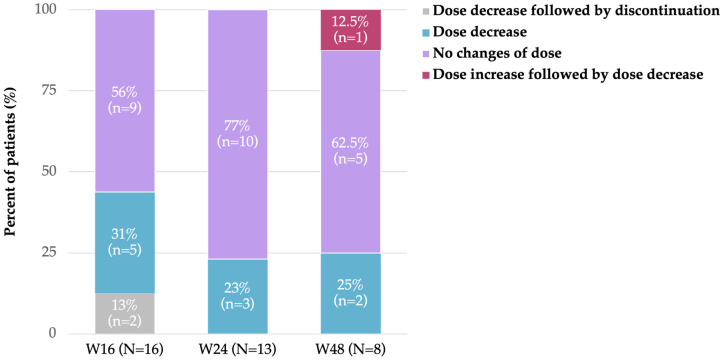
mOCS treatment patterns during benralizumab treatment (FAS). Abbreviations: mOCS, maintenance oral corticosteroid(s); N, number of patients; *n*, number of patients in a given category; and W, week.

**Figure 2:**
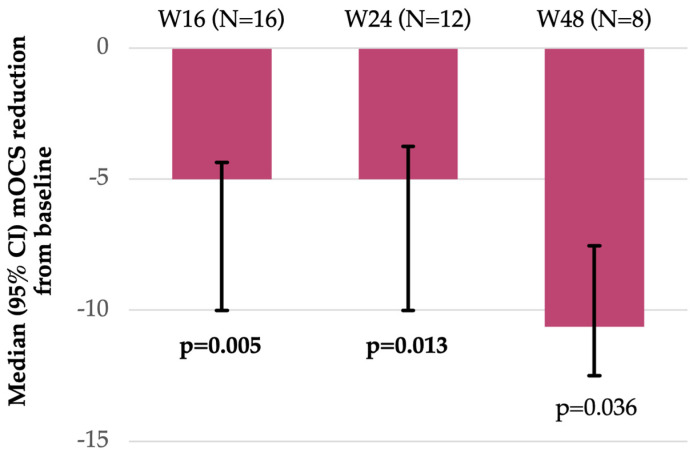
Changes in prednisone-equivalent daily dose from baseline up to 48 weeks after benralizumab initiation (FAS). Notes: Bolded values indicate statistical significance (Wilcoxon Signed-Rank test). Bonferroni correction: *p* < 0.0167. Abbreviations: CI, confidence interval; mOCS, maintenance oral corticosteroid(s); N, number of patients; and W, week.

During benralizumab treatment, and across the various timepoints, different type of changes in mOCS were applied, predominating dose decreases (Figure 1). The reduction in mOCS dose was statistically significant at W16 (*p* = 0.005) and W24 (*p* = 0.013) (Figure 2).

### 3.3. Clinical Outcomes During Treatment with Benralizumab

#### 3.3.1. Exacerbations

**Figure 3:**
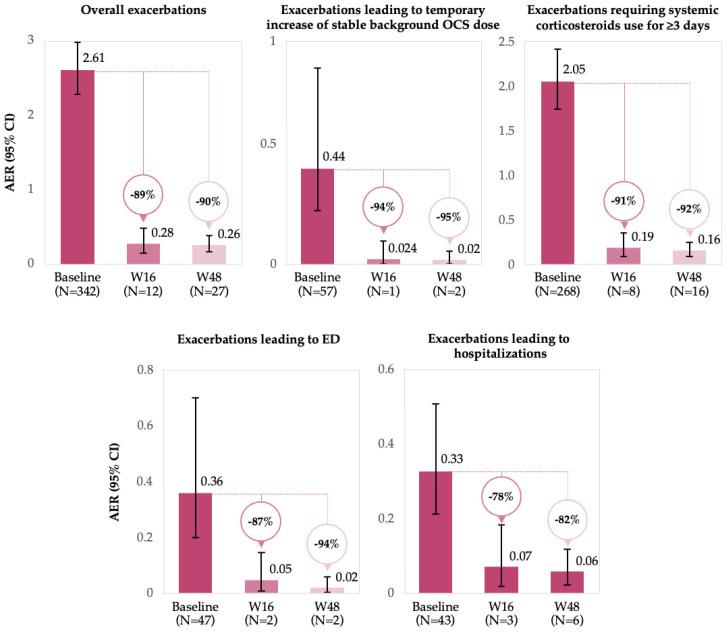
The relative reduction in AER from baseline to 16 and 48 weeks after benralizumab initiation for all categories of exacerbations (FAS). Abbreviations: AER, annualized exacerbation rate; CI, confidence interval; ED, emergency department; N, number of exacerbations; OCS, oral corticosteroid(s); and W, week.

At W16 and W48 after benralizumab treatment initiation, the total absolute number of exacerbation events reported was reduced to 12 and 27, respectively, as compared to 342 exacerbation events described at baseline. The overall AER (95% CI) was reduced from 2.61 (2.28–2.98) at baseline to 0.28 (0.15–0.49) at W16 and 0.26 (0.17–0.39) at W48, accounting for a relative reduction of 89% and 90%, respectively. A similar trend in AER reduction was observed across all categories of exacerbations, with a relative reduction ranging from 78 to 94% at W16 and 82–95% at W48 (Figure 3). All exacerbations that occurred during the follow-up period of interest were resolved without benralizumab discontinuation.

#### 3.3.2. Laboratory Parameters, Lung Function, and ACT Score

**Figure 4:**
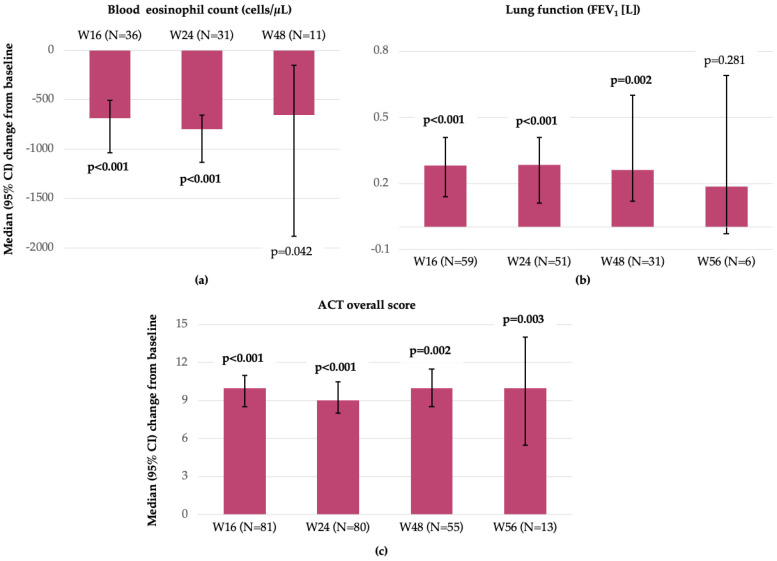
(**a**) Changes in blood eosinophil count during benralizumab treatment; (**b**) Changes in lung function during benralizumab treatment; and (**c**) Changes in ACT overall score during benralizumab treatment (FAS). Note: Bolded values indicate statistical significance (Wilcoxon Signed-Rank test); Bonferroni correction: *p* < 0.0167 for blood eosinophil count, *p* < 0.0125 for FEV_1_ and ACT score. Abbreviations: ACT, asthma control test; CI, confidence interval; FEV_1_, forced expiratory volume in one second; N, number of patients; and W, week.

During treatment with benralizumab, bEOS counts decreased from a median (IQR) of 620 (370–1030) cells/µL at index date to a median of 1 (0–30) cells/µL at W16 (*n* = 36; *p* < 0.001), 10 (0–50) cells/µL at W24 (*n* = 31; *p* < 0.001), and 6 (0–37.5) cells/µL at W48 (*n* = 11; *p* = 0.042) (Figure 4a). W56 bEOS data were excluded from the statistical analysis due to the low number of patients with available results (*n* = 4, median [IQR]: 0 [0;12.5]). Lung function improved across visits, changing from a median (IQR) FEV_1_ (L) of 1.78 (1.25–2.42) at index date to 2.06 (1.66–2.62) at W16 (*n* = 59; *p* < 0.001), 2.15 (1.51–2.61) at W24 (*n* = 51; *p* < 0.001), 1.96 (1.66–2.83) at W48 (*n* = 31; *p* = 0.002), and 2.68 (2.52–2.78) at W56 (*n* = 6; *p* = 0.281) (Figure 4b). FeNO measurement was limited to 1 patient at W16.

The median overall ACT score improved during benralizumab treatment, changing from a median (IQR) score of 11.5 (9–14.3) at baseline to 22 (18.5–24) at W16, 21 (19.0–24) at W24, 22 (20.0–24) at W48, and 20 (18.0–22) at W56. In patients with paired data available at specific timepoints, changes from baseline score showed improvements at all timepoints: W16 (*n* = 81; *p* < 0.001; +10.5 points), W24 (*n* = 80; *p* < 0.001; +9.5 points), W48 (*n* = 55; *p* = 0.001; +10.5 points), and W56 (*n* = 6; *p* = 0.003; +8.5 points) (Figure 4c). Overall, the percentage of patients with asthma control (ACT > 19) increased during benralizumab treatment, ranging from 54% to 76% over the 56-week follow-up period compared to baseline, when only 2.3% of patients reported controlled asthma (ACT score >20).

## 4. Discussion

We described the baseline clinical characteristics and treatment patterns of a cohort of 131 patients with SEA who received benralizumab as an add-on therapy in real-life settings in Romania as part of the non-interventional study BREEZE. Our findings support benralizumab effectiveness in reducing mOCS use, decreasing exacerbations, depleting bEOS, and improving lung function and asthma control. To date, this represents the first real-world data on benralizumab use in SEA patients in Romania and provides clinicians and payers with more recent insights into its use in routine clinical settings, beyond clinical trials.

The effectiveness results in the Romanian cohort of the BREEZE study are consistent with those of the overall study (CEE-BA countries: N = 381) [15,16] and other XALOC-1 studies, such as ANANKE (Italy, *n* = 205) [11], BETREAT (Portugal, *n* = 74) [12], BPAP (UK, *n* = 208) [13], and ORBE-II (Spain, *n* = 204) [14], and also other studies not part of the XALOC-1 program, such as PROMISE (Belgium, *n* = 73) [17], a single-center recent study from Central Eastern Europe (Poland, *n* = 103) [18], and ZEPHYR2 (US, insurance claims analysis, eosinophilic cohort *n* = 429) [19]. Data from these studies support the beneficial effects of benralizumab in SEA and demonstrate sustained improvement of clinical outcomes over time.

In our cohort, over half (66%) of patients were females, with a late onset of asthma (mean [SD] age of asthma onset 42.8 [15.3] years), a long-term disease at benralizumab initiation (mean [SD] duration of asthma 11.6 [11] years), and a high rate of comorbidities (91%), with chronic rhinitis and allergies being the most common (73% and 53% of patients, respectively). Before benralizumab initiation, this cohort described frequent exacerbations (mean [SD]: 2.6 [2.2] per year), poor symptom control (81% of patients with ACT < 16), high blood eosinophil counts (90% of patients with bEOS ≥ 300 cells/μL), and mOCS use in 1 in 5 patients. This patient profile is representative of SEA and similar to most observed in other real-world cohorts from the CEE-BA countries [16], Italy [11], Portugal [12], Spain [14], and the US [19]. Exceptions were noted for the age at onset, which was lower in UK patients (31.2 [18.9] years) [13]; for the disease duration at benralizumab initiation, which was longer in CEE-BA patients (16.9 [13.6] years) [16], probably due to late reimbursement, and also in Spanish patients (15.1 [12.7] years) [14] and Belgian patients (18.4 years) [17]; and for the comorbidity rate at baseline, which was lower in Italian patients (50.2%) [11] and UK patients (69%) [13]. Lastly, in a previous study assessing the treatment eligibility for biologics before the COVID-19 pandemic in SEA patients in Romania [20], we observed similar demographic characteristics and chronic mOCS use, showing thus a trend of stability over time of the patient profile in our country.

In the Romanian cohort of the BREEZE study, about 1 in 5 SEA patients were using mOCS (85% received high doses of >5 mg of prednisone-equivalent daily) at baseline to manage asthma-related symptoms and prevent exacerbations, although the long-term use of OCSs remains a concern due to their associated adverse effects [21]. Within the other studies included in the XALOC-1 program, the number of patients on regular mOCS treatment differed: in the CEE-BA cohort it was similar to Romania (around 1 in 5 patients), but it was higher in the Italian (around 1 in 4 at index date), Spanish (around 1 in 3), and UK (around 1 in 2) cohorts [11,13,14,16]. This difference may be attributed to variations in demographic groups and routine practices across European countries. In the Romanian dataset, around one-quarter of patients experienced a dose reduction over time, with a median reduction of daily dose (prednisone-equivalent) from baseline to W16 of 5 (95% CI: 4.37–10) mg/day (*p* = 0.005) and from baseline to W24 of 5 (95% CI: 3.75–10) mg/day (*p* = 0.0013), respectively. These findings complement, and extend to a new geography, the results of clinical trials and other real-world studies from the XALOC-1 program that showed significant reductions in mOCS intake during treatment with benralizumab [11,12,13,14,17,19,22]. In a study from The Netherlands (RAPSODI), mOCS dosage and its decrease of ≥50% over 3 months were taken into account together with baseline patient characteristics (sex, prior biologic usage, bEOS, and FEV_1_) and a 3-month Asthma Control Questionnaire (ACQ-6) to predict long-term (1 year) responders to benralizumab [23]. Such tools could prove useful to clinicians in tailoring individualized treatments and thus facilitating the effective use of biologic therapies.

During treatment with benralizumab, we observed improvements in asthma exacerbation rate, consistent with findings from the CEE-BA countries [16], XALOC-1 program, and other real-world studies [11,12,13,14,17,18,19,24]. The exacerbation rate declined by approximately 90%, decreasing from an initial AER of 2.61 to 0.28 at W16 and 0.26 at W48, and all exacerbations occurring while on benralizumab were resolved without requiring its discontinuation. These findings align with those reported in the CEE-BA countries, the Italian and UK cohorts, and the pooled data from XALOC-1, in which the decline in exacerbation rates ranged between 81% and 93% at similar timepoints for the overall population [10,11,13,16,17,24]. Other real-world studies reported an 87% reduction in severe exacerbations and a 52–64% reduction in overall exacerbations at 1 year [14,19].

In our cohort, the bEOS counts decreased as early as W16 and W24 (*p* < 0.001). The unique mechanism of action of benralizumab, targeting IL-5Rα and inducing eosinophil apoptosis via antibody-dependent cytotoxicity, leads to complete eosinophil depletion in SEA patients [25]. This specific effect of benralizumab was shown to be linked with clinically meaningful reductions in airway hyperresponsiveness among SEA patients, which could further contribute to improvements in asthma control and quality of life in these patients [26]. In our study, asthma control improved over time, with stable median increases in ACT score ranging between 9.5 and 10.5 units between W16 and W48 in patients with available data. These results align with the findings on asthma control of other real-world studies [11,13,14,16]. While in our study, the number of patients with paired FEV_1_ data available across various timepoints reduced significantly by W56, on the small datasets, we observed significant improvement over time, which should be further explored and validated in our population. Across the XALOC-1 studies [11,14,16], a significant post-bronchodilator response (≥200 mL) [27] was seen, a consistent finding that should be explored in more depth in longer-term follow-up studies. In addition, it is important to corroborate clinical results with the patient perspective and expectations from their therapies in the contemporary treatment landscape, as all these factors could provide a more comprehensive understanding of the local healthcare context and directions to maximize treatment effectiveness.

Only a few discontinuation events were reported in this cohort, which is a very encouraging finding; however, longer follow-up periods are required to better understand the disease course under this biologic therapy. At the same time, it is important to remark that delays in administering injections during routine visits were observed, attributed mainly to the COVID-19 pandemic that overlapped with the period of interest for this study and impacted the availability of benralizumab in hospital or outpatient clinic pharmacies. Nonetheless, this does not seem to affect patient outcomes.

In the Romanian cohort, only 7% of patients reported a previous biologic use, similar to the CEE-BA cohort (9%), but substantially lower compared to Italian (28%), Portugal (32%), UK (43%), and Spanish (31%) cohorts [11,12,13,14,15,16]. In Romania, this may be partly due to the late reimbursement as compared to Western countries, but also due to an initial hesitation in prescribing biologics for SEA, as highlighted by a local study in severe uncontrolled asthma patients (*n* = 28) receiving biologics (omalizumab, benralizumab, and dupilimumab) [28]. However, the improved patient outcomes observed in our study emphasize the significant clinical benefits of benralizumab for SEA patients, advocating for the broader use of biologics to achieve more rapid clinical improvement and reduce mOCS use.

The main limitations of this study are related to its real-world design and include site selection, reduced availability of biological and spirometry data, and variability in treatment practices, which may have impacted the generalizability of our findings. The COVID-19 pandemic also affected the spirometry and routine tests that SEA patients perform in real-life settings, as the time allocated for visits was reduced and some hospitals even had interdictions to perform spirometry as a measure to reduce the exposure of patients to Severe Acute Respiratory Syndrome Coronavirus 2 (SARS-CoV-2). While in other countries, home use of benralizumab was encouraged specifically in this regard, this was not applicable at that time for Romanian patients. A different and more robust methodology addressing the lack of a specific period for following up the entire population along with higher sample sizes at each timepoint should be employed in future research to validate the preliminary results observed in this cohort. The current design has impacted the evaluation of outcomes in a homogenous manner and the direct comparisons with other similar studies. The longitudinal analyses are exploratory and further limited by the variable sample sizes, decreasing over time and affecting the interpretability of findings, which could not be seen as true longitudinal effects. Due to the funneling approach of the study and the reduced number of patients with paired data available over time, the results should be interpreted with caution. Due to the limited number of patients over time, subgroups of clinical interest could not be performed. Larger datasets and longer follow-up periods are needed in future studies, to address relevant questions for the local medical community, such as rates of clinical remission over time and predictors of benralizumab response in the patient population from Romania.

Under specific circumstances, the real-world design could be considered a strength. BREEZE allowed for data gathering in a centralized manner from many (*n* = 23) inpatient and outpatient clinical centers across Romania, being representative of the SEA patients treated with benralizumab for up to one year in our country. Real-world evidence studies are becoming increasingly important to regulatory agencies to inform decision-making processes for new indications/label expansion across various therapeutic areas, as well as for reimbursement authorities [29].

## 5. Conclusions

To our knowledge, this is among the first real-world studies built on a large cohort of patients with SEA receiving benralizumab for up to 1 year in day-to-day practice in Romania. The findings from our national cohort contribute to the increasing data on the effectiveness of benralizumab among patients with SEA, as shown by meaningful reductions in exacerbations and mOCS use, and improvement of asthma control over time. Further longer-term, broader studies are needed to validate our preliminary findings in the benralizumab patient population from Romania.

## Figures and Tables

**Table 1:** Demographic characteristics of SEA patients at baseline (FAS).

Characteristics	FAS (N = 131)
Age at index date, years	
Mean (SD)	54.4 (12.4)
Age at asthma onset, years	
Mean (SD)	42.8 (15.3)
Female patients, *n* (%)	86 (65.7)
Time since asthma diagnosis at index date, years	
Mean (SD)	11.6 (11)
BMI, kg/m^2^	
Mean (SD)	28.5 (7.03)
Patients with BMI > 25 kg/m^2^, *n* (%)	82 (62.6)
Smoking history, *n* (%)	
Current smoker	10 (7.6)
Former smoker	32 (24.4)
Never smoker	89 (67.9)
Asthma-related comorbidities, *n* (%)	
At least one comorbidity	119 (90.8)
Chronic rhinitis	95 (72.5)
Allergies	69 (52.7)
Respiratory infections	60 (45.8)
Nasal polyposis	40 (30.5)
Bronchiectasis	28 (21.4)
Gastro-esophageal reflux	15 (11.5)
Other *	21 (16.0)
OCS-related comorbidities, *n* (%)	
At least one comorbidity	57 (43.5)
Systemic hypertension	38 (29.0)
Cardiovascular disease	24 (18.3)
Obesity/metabolic syndrome	20 (15.3)
Hypercholesterolemia	17 (13.0)
Dyspeptic disorders	14 (10.7)
Osteoporosis/osteopenia	13 (9.9)
Type 2 diabetes mellitus	7 (5.3)
Sleep disorders	6 (4.6)
Other **	11 (8.4)
COVID-19, *n* (%)	
COVID-19 occurrence	27 (20.6)
COVID-19 leading to hospitalization, *n* (%)	4 (3.1)

* Other included asthma-COPD overlap (5 patients), atopic dermatitis (2 patients), and other non-specified asthma-related comorbidities (14 patients); ** Other included psychiatric disorders (4 patients), myopathies (2 patients), and other non-specified OCS-related comorbidities (5 patients). Abbreviations: BMI, body mass index; COVID-19, coronavirus disease 2019; FAS, full analysis set; OCS, oral corticosteroid(s); IQR, interquartile range; N, total number of patients; *n* (%), number (percent) of patients in a given category; SEA, severe eosinophilic asthma; and SD, standard deviation.

**Table 2:** SEA characteristics at baseline (FAS).

Characteristics	FAS (N = 131)
Biomarkers *	
bEOS count, cells/μL	
Number of patients	130
Median (IQR)	620 (370–1031)
bEOS 300–400, *n* (%)	24 (18.5)
bEOS ≥ 400, *n* (%)	94 (72.3)
Total IgE, IU/mL	
Number of patients	61
Median (IQR)	143 (68–445)
Lung function	
FEV_1_ at index, L	
Number of patients	106
Median (IQR)	1.8 (1.3–2.4)
Asthma control	
ACT score	
Number of patients	128
Mean (SD)	11.7 (4.15)
ACT < 16, *n* (%)	103 (80.5)
Exacerbations	
Exacerbations	
At least one exacerbation, *n* (%)	116 (88.6)
Mean (SD)	2.6 (2.2)
AER (95% CI)	2.61 (2.28–2.98)
Healthcare resource utilization	
Healthcare resource utilization (asthma-related), *n* (%)	
ED visits	19 (14.5)
ICU hospitalizations	1 (0.8)
Non-ICU hospitalizations	29 (22.1)
Treatments	
mOCS	
Patients with baseline mOCS, *n* (%)	27 (20.6)
Prednisone-equivalent daily dosage (mg/day), median (IQR)	15 (10–20)
Patients with >5 mg/day **, *n* (%)	23 (85.2)
Other respiratory treatments, *n* (%)	
Combined ICS/LABA	131 (100)
ICS ***	7 (5.3)
LABA ***	1 (0.8)
LAMA	45 (34.4)
LTRA	86 (65.7)
Theophylline	13 (9.9)
Previous biologic treatments ^#^, *n* (%)	9 (6.9)

* FeNO results were available only for 2 patients, with a median (IQR) of 10 (9–11). ** Reported among patients who used OCS at baseline; *** ICS and LABA monotherapy; ^#^ Omalizumab was received prior to benralizumab initiation. Abbreviations: ACT, asthma control test; AER, annualized exacerbation rate; bEOS, blood eosinophils; CI, confidence interval; ED, emergency department; FAS, full analysis set; FeNO, Fractional exhaled Nitric Oxide; FEV_1_, forced expiratory volume in one second; ICS, inhaled corticosteroid; ICU, intensive care unit; IgE, immunoglobulin E; IQR, interquartile range; IU, international units; LABA, long-acting beta_2_-agonists; LAMA, long-acting muscarinic agonists; LTRA, leukotriene receptor agonists; mOCS, maintenance oral corticosteroid(s); N, total number of patients; *n* (%), number (percent) of patients in a given category; ppb, parts per billion; and SD, standard deviation.

**Table 3:** Treatments at index date and during benralizumab therapy (FAS).

Treatment *n* (%)	Index date (N = 131)	W4 (N = 129)	W8 (N = 123)	W16 (N = 116)	W24 (N = 106)	W32 (N = 97)	W40 (N = 90)	W48 (N = 70)	W56 (N = 13)
Benralizumab	131 (100)	129 (100)	123 (100)	116 (100)	106 (100)	97 (100)	90 (100)	70 (100)	13 (100)
mOCS	20 (15.3)	21 (16.3)	17 (13.8)	16 (13.8)	13 (12.3)	11 (11.3)	10 (11.1)	8 (11.4)	0
ICS *	5 (3.8)	6 (4.7)	5 (4.1)	4 (3.5)	3 (2.8)	3 (3.1)	3 (3.3)	3 (4.3)	0
ICS/LABA	130 (99.2)	129 (100)	123 (100)	116 (100)	106 (100)	97 (100)	90 (100)	70 (100)	12 (92.3)
LAMA	41 (31.3)	41 (31.8)	41 (33.3)	38 (32.8)	37 (34.9)	30 (30.9)	27 (29.7)	21 (30.0)	2 (15.4)
LTRA	82 (62.6)	82 (63.6)	76 (61.8)	67 (57.8)	57 (53.8)	48 (49.5)	48 (52.8)	35 (50.0)	6 (46.2)
Theophylline	9 (6.9)	10 (7.8)	11 (8.9)	11 (9.5)	11 (10.4)	8 (8.3)	8 (8.8)	8 (11.4)	1 (7.7)

* ICS as monotherapy. No patient received LABA as monotherapy after index date. Abbreviations: FAS, full analysis set; ICS, inhaled corticosteroid; LABA, long-acting beta_2_-agonists; LAMA, long-acting muscarinic agonists; LTRA, leukotriene receptor agonists; mOCS, maintenance oral corticosteroid(s); N, total number of patients; and *n* (%), number (percent) of patients in a given category.

## Data Availability

The datasets collected and analyzed during the study are available from the corresponding author upon reasonable request. Data underlying the findings described in this manuscript may be obtained in accordance with AstraZeneca’s data sharing policy described at https://astrazenecagrouptrials.pharmacm.com/ST/Submission/Disclosure, accessed on 21 January 2025.

## Supplementary Materials

The following supporting information can be downloaded at: https://www.mdpi.com/article/10.3390/jcm15020425/s1, Table S1: Reimbursement criteria for benralizumab as add-on maintenance therapy in severe eosinophilic asthma applicable in Romania

## Abbreviations

The following abbreviations are used in this manuscript:

ACT	Asthma control test
AER	Annualized exacerbation rate
bEOS	Blood eosinophils
BMI	Body mass index
CEE-BA	Central Eastern Europe and Baltic Area
CI	Confidence intervals
COVID-19	Coronavirus disease 2019
ED	emergency department
FAS	Full analysis set
FcεRI	High-affinity receptor for IgE
FeNO	Fractional exhaled nitric oxide
FEV_1_	Forced expiratory volume in one second
ICS	Inhaled corticosteroids
ICU	Intensive care unit
IgE	Immunoglobulin E
IL	Interleukin
IL-5(R)	Interleukin-5 (receptor)
IQR	Interquartile range
LABA	Long-acting beta2-agonists
LAMA	Long-acting muscarinic agonists
LTRA	Leukotriene receptor agonists
mOCS	Maintenance oral corticosteroid(s)
(μ) L	(Micro) liter
SARS-CoV-2	Severe Acute Respiratory Syndrome Coronavirus 2
SEA	Severe eosinophilic asthma
SD	Standard deviation
W	week
