# Supplementary material for: Real-World Effectiveness Following Benralizumab Use in Patients with Severe Eosinophilic Asthma in Romania: A Retrospective Cohort Study (BREEZE)
Source: J Clin Med. 2026 Jan 6;15(2):425. doi: 10.3390/jcm15020425 (PMC12841929; doi:10.3390/jcm15020425)
Supplement: Supplementary file 1 [file jcm-15-00425-s001.zip › jcm-4015084-supplementary.pdf]

Supplemental material

Table S1. Reimbursement criteria for benralizumab as add-on maintenance therapy in severe eosinophilic asthma applicable in Romania

**Reimbursement criteria for benralizumab**
• **Age** over 18 years (adults)<br>• **Diagnosis of severe asthma**, according to the recommendations of the Global Strategy for Asthma Management and Prevention (GINA).<br>• **Eosinophils** in peripheral blood: ≥ 300 cells / µL at the start of treatment or ≥ 150 cells / µL in those treated intermittently or continuously with OCS ≥ 8 mg / day (8 mg prednisone / equivalent 6 mg methylprednisolone); EOS tests results should not exceed the last 12 months.<br>• **Management of asthma prescribed by a specialist doctor,** with a follow-up period of at least 6 months, including:<br>a) treatment with high-dose ICS, in combination with a LABA for at least 6 months (correct inhalation technique and adherence to treatment confirmed by the attending physician);<br>b) correct management of comorbidities<br>• **Lack of asthma control, according to the GINA guideline**, defined by one of:<br>a) reduced control of symptoms (frequent symptoms or frequent use of symptom relief therapy, limited asthma activity, nocturnal awakenings caused by asthma);<br>b) frequent exacerbations (≥2 / year) requiring short-term oral and / or injectable corticosteroids, or ≥1 exacerbation / year in those with daily OCS, or severe exacerbations (≥1 / year) requiring hospitalization.

Abbreviations: EOS, eosinophils; GINA, Global Strategy for Asthma Management and Prevention; ICS, inhaled corticosteroids; LABA, long-acting $beta_2$-agonists; OCS, oral corticosteroids.
